# Reports of Forgone Medical Care Among US Adults During the Initial Phase of the COVID-19 Pandemic

**DOI:** 10.1001/jamanetworkopen.2020.34882

**Published:** 2021-01-21

**Authors:** Kelly E. Anderson, Emma E. McGinty, Rachel Presskreischer, Colleen L. Barry

**Affiliations:** 1 Department of Health Policy and Management, Johns Hopkins Bloomberg School of Public Health, Baltimore, Maryland

## Abstract

**Importance:**

The coronavirus disease 2019 (COVID-19) pandemic has caused major disruptions in the US health care system.

**Objective:**

To estimate frequency of and reasons for reported forgone medical care from March to mid-July 2020 and examine characteristics of US adults who reported forgoing care.

**Design, Setting, and Participants:**

This survey study used data from the second wave of the Johns Hopkins COVID-19 Civic Life and Public Health Survey, fielded from July 7 to July 22, 2020. Respondents included a national sample of 1337 individuals aged 18 years or older in the US who were part of National Opinion Research Center’s AmeriSpeak Panel.

**Exposures:**

The initial period of the COVID-19 pandemic in the US, defined as from March to mid-July 2020.

**Main Outcomes and Measures:**

The primary outcomes were missed doses of prescription medications; forgone preventive and other general medical care, mental health care, and elective surgeries; forgone care for new severe health issues; and reasons for forgoing care.

**Results:**

Of 1468 individuals who completed wave 1 of the Johns Hopkins COVID-19 Civic Life and Public Health Survey (70.4% completion rate), 1337 completed wave 2 (91.1% completion rate). The sample of respondents included 691 (52%) women, 840 non-Hispanic White individuals (63%), 160 non-Hispanic Black individuals (12%), and 223 Hispanic individuals (17%). The mean (SE) age of respondents was 48 (0.78) years. A total of 544 respondents (41%) forwent medical care from March through mid-July 2020. Among 1055 individuals (79%) who reported needing care, 544 (52%) reported forgoing care for any reason, 307 (29%) forwent care owing to fear of severe acute respiratory syndrome coronavirus 2 (SARS-CoV-2) transmission, and 75 (7%) forwent care owing to financial concerns associated with the COVID-19 pandemic. Respondents who were unemployed, compared with those who were employed, forwent care more often (121 of 186 respondents [65%] vs 251 of 503 respondents [50%]; *P* = .01) and were more likely to attribute forgone care to fear of SARS-CoV-2 transmission (78 of 186 respondents [42%] vs 120 of 503 respondents [24%]; *P* = .002) and financial concerns (36 of 186 respondents [20%] vs 28 of 503 respondents [6%]; *P* = .001). Respondents lacking health insurance were more likely to attribute forgone care to financial concerns than respondents with Medicare or commercial coverage (19 of 88 respondents [22%] vs 32 of 768 respondents [4%]; *P* < .001). Frequency of and reasons for forgone care differed in some instances by race/ethnicity, socioeconomic status, age, and health status.

**Conclusions and Relevance:**

This survey study found a high frequency of forgone care among US adults from March to mid-July 2020. Policies to improve health care affordability and to reassure individuals that they can safely seek care may be necessary with surging COVID-19 case rates.

## Key Points

**Question:**

What are the frequency of and reasons for reported forgone medical care from March to mid-July 2020, the initial phase of the coronavirus disease 2019 (COVID-19) pandemic in the US?

**Findings:**

In this national survey of 1337 participants, 41% of respondents reported forgoing medical care from March through mid-July 2020. Among adults who reported needing care during this period, more than half reported forgoing care for any reason, more than one-quarter reported forgoing care owing to fear of severe acute respiratory syndrome coronavirus 2 transmission, and 7% reported forgoing care owing to financial concerns.

**Meaning:**

This survey study found that there was a high frequency of forgone care from March to mid-July 2020, with respondents commonly attributing the causes of forgone care to repercussions of the COVID-19 pandemic.

## Teaser

This survey study examines the frequency of and reasons for reported forgone care among US adults during the COVID-19 pandemic.

## Introduction

During the initial months of the coronavirus disease 2019 (COVID-19) pandemic, the US health care system experienced major disruptions, with temporary closures of medical practices, cancellation of elective procedures, and the shift of many services to telehealth delivery.^1^ These disruptions may have led individuals to forgo medical care. Forgoing care for chronic and emergent conditions can lead to increased complications and costs. Additionally, missing preventive care, such as cancer screenings, can result in a delayed diagnosis. Since the pandemic onset, hospitals have reported substantial declines in emergency department (ED) visits for severe health issues, including heart attacks and strokes.^2^

Several factors may have influenced individuals’ decisions to forgo medical care during the COVID-19 pandemic. In March 2020, many state and local governments issued emergency public health orders, such as stay-at-home orders and bans on elective procedures, which either discouraged or prohibited certain types of medical care.^1^ These suspensions were not lifted until late spring or early summer 2020. Furthermore, many medical practices voluntarily closed in the early weeks of the pandemic, either to redirect their personnel to COVID-19 response or to reduce risk of transmission of the severe acute respiratory syndrome coronavirus 2 (SARS-CoV-2), the virus that causes COVID-19. Many individuals feared that seeking in-person medical care could expose them to SARS-CoV-2.

In addition, the financial downturn caused by the COVID-19 pandemic increased unemployment rates and reduced employee working hours. In the first 4 months of the pandemic, more than 48 million individuals filed for unemployment benefits.^3^ Because health insurance is tied to employment for many US adults, layoffs have also resulted in more than 12 million individuals losing coverage since March 2020.^4^ Resulting financial concerns may have influenced individuals’ decisions to obtain or forgo care.

Several studies have sought to quantify changes in medical care during the pandemic using electronic health record (EHR) or insurance claims data. A study by Westgard et al^5^ found a 49% decline in ED visits comparing visits in the 28 days before and 28 days after the state emergency declaration using EHR data from an urban trauma center.^5^ Using data from 9 cardiac catheterization laboratories, a study by Garcia et al^6^ estimated a 38% decline in cardiac catheterizations, comparing data from March 2020 with data from 2019 and earlier in 2020. Similarly, a study by Bhatt et al^7^ estimated a 43% reduction in hospitalizations for cardiovascular conditions in March 2020 compared with March 2019, using data from a large health system. While these studies provide a useful snapshot of changes in health care utilization, they do not provide a nationally representative picture of forgone care or assess the mechanisms behind reductions in care. Understanding reasons individuals forgo care is particularly important for designing clinical and policy interventions targeted to barriers to obtaining care. Furthermore, these prior studies focused on care for severe health issues and did not examine preventive care, mental health care, or prescription medication continuity.

To our knowledge, no published research has quantified the frequency of and factors associated with forgone medical care during the initial phase of the COVID-19 pandemic in a representative sample of US adults. We fielded a nationally representative survey to determine the frequency and types of forgone medical care among adults and the reasons identified for cancelling or not seeking care from March through mid-July 2020. We examined the sociodemographic characteristics of respondents forgoing medical care and assessed whether prevalence differed for certain at-risk groups, including individuals who were unemployed, lacked health insurance, or had chronic health conditions. Finally, we examined 2 specific reasons respondents may have forgone medical care: fear of exposure to SARS-CoV-2 and the financial repercussions of the COVID-19 pandemic.

## Methods

All data reported in this survey study come from wave 2 of the Johns Hopkins COVID-19 Civic Life and Public Health Survey, fielded July 7 to 22, 2020, using the National Opinion Research Center’s (NORC) AmeriSpeak Panel. Prior to enrolling individuals in the AmeriSpeak Panel, NORC obtained written informed consent. This study was approved by the Johns Hopkins Bloomberg School of Public Health institutional review board. This study is reported following the American Association for Public Opinion Research (AAPOR) reporting guideline.

The AmeriSpeak Panel is a probability-based panel designed to be representative of the US adult population. The panel is drawn from NORC’s area probability sample and US Postal Service addresses and covers 97% of US households.^8^ The AmeriSpeak panel’s recruitment rate is 34% and includes approximately 35 000 individuals. Our sample was drawn from this panel, and respondents completed the survey online.

We developed a 16-item module to assess health status and forgone medical care from March to the time of survey data collection in July 2020 (eAppendix in the Supplement). Possible types of forgone medical care included missed prescription medications, missed scheduled preventive care visits, missed scheduled general medical outpatient visits (ie, physical health care, other than preventive care, delivered in an office setting), missed scheduled mental health outpatient visits, missed elective surgical procedures, or emergent health issues warranting general medical or mental health care for which the respondent did not receive care. In the survey, we asked respondents to distinguish between care received through telehealth (not classified as forgone care) and missed or forgone care. We defined a new health issue as severe if a respondent reported a severity score of 4 or 5 on a 5-point Likert scale. In addition to the aggregate measure that included all of the categories of forgone care, we also developed a measure of forgone planned medical care that included prescription medications, scheduled preventive care visits, scheduled general medical outpatient visits, scheduled mental health outpatient visits, and elective surgical procedures but did not include new health issues.

We calculated prevalence of forgone medical care overall and by type of care among all respondents and among the subset who reported needing care. Then, among individuals who reported needing care, we calculated prevalence of forgone medical care by sociodemographic and clinical characteristics and tested whether group differences were statistically significant. We also analyzed group differences based on race/ethnicity, as the COVID-19 pandemic has disproportionately affected Black, Hispanic, and Indigenous communities.^9,10,11^ We classified individual race/ethnicity based on self-reported race/ethnicity using response options defined by NORC. Finally, we tested whether frequency of forgone medical care differed by employment and health insurance status.

### Statistical Analysis

All counts and percentages reported in this study are survey weighted To test whether frequency of forgone medical care differed between subgroups, we used Pearson χ^2^ tests. We considered a difference to be statistically significant if the 2-sided *P* value was less than .05. We conducted analyses in Stata statistical software version 16 (StataCorp), applying survey weights to calculate nationally representative estimates. Data were analyzed from July 30 to September 3, 2020.

## Results

Of 1468 individuals who completed wave 1 of the survey (70.4% completion rate), 1337 completed wave 2 (91.1% completion rate). Among 1337 wave 2 respondents, 691 (52%) were women, and the mean (SE) age was 48 (0.78) years. A total of 840 respondents (63%) reported their race/ethnicity as non-Hispanic White, 160 respondents (12%) reported their race/ethnicity as non-Hispanic Black, 223 respondents (17%) reported their race/ethnicity as Hispanic, and the remaining 115 respondents (9%) reported another race and non-Hispanic ethnicity (eTable in the Supplement).

**Figure 1.**
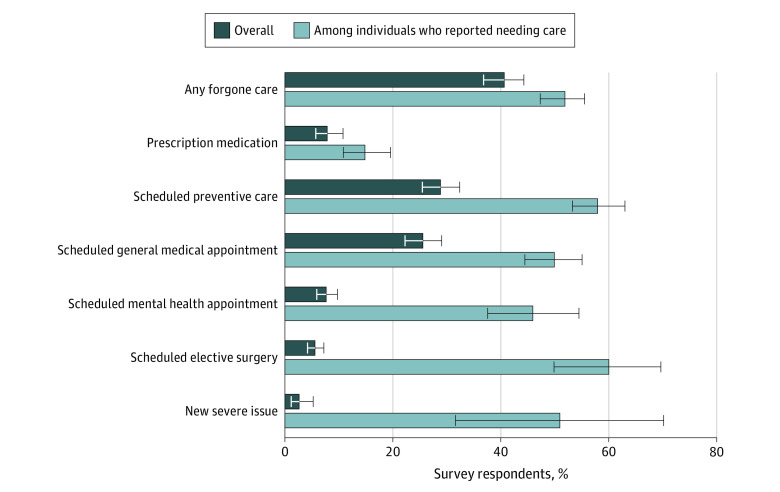
Share of Respondents Forgoing Medical Care From March Through Mid-July 2020 Forgone medical care includes missing 1 or more doses of a medicine the respondent typically picked-up or had someone else pick-up from a retail pharmacy; missing a scheduled health care visit, including a preventive care visit, general medical outpatient visit, mental health outpatient visit, or elective surgical procedure; or not receiving care for a new severe (defined based on self-report as severity 4-5 on a scale of 1-5) physical or mental health issue. Individuals could report multiple types of forgone care during the period of March through mid-July 2020.

A total of 544 respondents, representing an estimated 41% of US adults , reported forgoing medical care during the initial phase of the COVID-19 pandemic in the US from March through mid-July 2020 (Figure 1), including 108 respondents (8%) who reported missing 1 or more doses of a prescription medicine typically picked up from a retail pharmacy, 387 respondents (29%) who reported missing a preventive care visit, 343 respondents (26%) who reported missing an outpatient general medical appointment, 105 respondents (8%) who reported missing an outpatient mental health appointment, 77 respondents (6%) who reported missing an elective surgery, and 38 respondents (3%) who reported not receiving health care for a new severe mental or physical health issue.

Among 1055 respondents (79%) who reported needing care from March to mid-July 2020, 544 (52%) reported forgoing care, including 108 of 725 respondents (15%) who typically picked up prescription medication and who missed 1 or more doses, 387 of 664 respondents (58%) with scheduled preventive care, 343 of 688 respondents (50%) with scheduled general medical care, and 105 of 227 respondents (46%) with scheduled mental health care reporting missing visits. Among 127 respondents who had scheduled an elective surgical procedure in the initial phase of the pandemic, 77 respondents (60%) reported forgoing their surgical procedure. Finally, 38 of 74 respondents (51%) with a severe mental or physical health issue that emerged after the start of the pandemic reported forgoing care.

**Figure 2.**
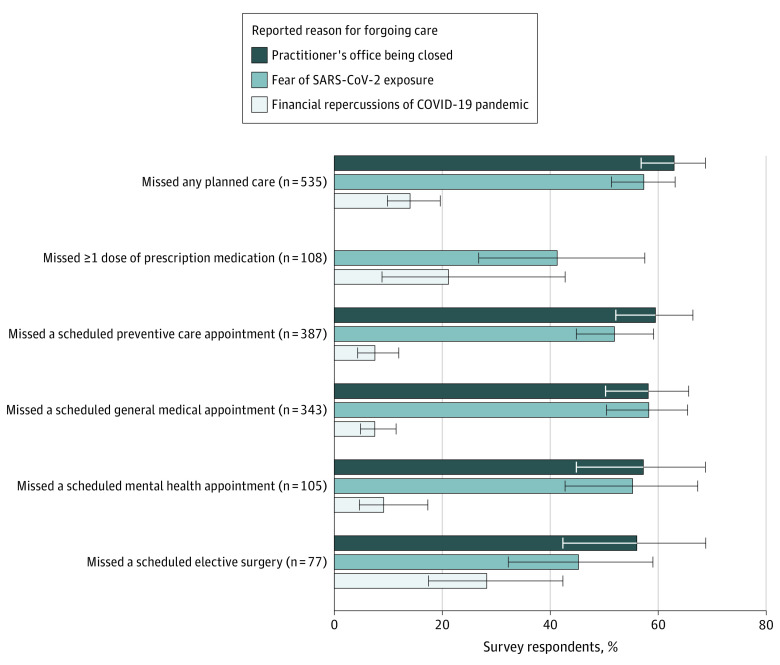
Reasons Reported for Forgoing Care Among Respondents Who Missed Planned Care From March Through Mid-July 2020 Respondents were prompted to select the reasons that best described why they missed taking a dose(s) of medication or missed a previously scheduled health care appointment. Respondents were allowed to select more than 1 reason. Practitioner practice being closed was not a response option for individuals who reported missing a dose of prescription medication. COVID-19 indicates coronavirus disease 2019; SARS-CoV-2, severe acute respiratory syndrome coronavirus 2.

Among 535 respondents who reported missing any planned medical care, including missed prescription medications or missed scheduled appointments or procedures, 337 (63%) attributed missed care to a medical practice being closed (either temporarily or permanently), 307 (57%) attributed missed care to fear of SARS-CoV-2 exposure, and 75 (7%) attributed missed care to financial repercussions of the COVID-19 pandemic (Figure 2). While medical practice closure was the most common reason for missing care, 174 respondents (56%) who reported missing care owing to fear of SARS-CoV-2 exposure and 39 respondents (52%) who reported missing planned care owing to the financial repercussions of the COVID-19 pandemic did not report medical practice closure as a reason for forgoing care.

Among 108 respondents reporting a missed dose of medication, 44 respondents (41%) attributed it to fear of COVID-19 and 23 respondents (21%) cited financial repercussions of the COVID-19 pandemic. Among 387 respondents who reported missing scheduled preventive care, scheduled general medical care, or scheduled mental health care, more than half of respondents attributed the missed care to practice closure and fear of COVID-19 exposure, and less than 10% of respondents attributed the forgone care to financial concerns owing to COVID-19 (Figure 2). Practice closure and fear of SARS-CoV-2 transmission were also the most common reasons reported for missing a scheduled elective surgery; more than one-quarter of respondents reported the financial repercussions of the COVID-19 pandemic as a reason for forgoing elective surgery (Figure 2).

While the proportion of respondents reporting forgone medical care did not vary by sex, differences were found by race/ethnicity, age, household income, employment status, and health insurance status (Table 1). A larger share of Hispanic respondents reported missed prescription medications compared with non-Hispanic White respondents (33 of 109 respondents [30%] vs 50 of 482 respondents [10%]; *P* = .004). Compared with adults aged 65 years or older, higher proportions of respondents reported missed medication in age groups 18 to 34 years (45 of 204 respondents [22%] vs 10 of 160 respondents [6%]; *P* = .004) and 35 to 49 years (29 of 182 respondents [16%]; *P* = .01). Respondents in households with lower incomes (ie, <$35 000/year) more often reported missing medication compared with respondents in households with an income of $35 000 to $74 999 per year (66 of 244 respondents [27%] vs 26 of 226 respondents [12%]; *P* = .01).

**Table 1.** Respondents Who Reported Needing Care Reporting Forgone Medical Care From March Through Mid-July 2020, by Sociodemographic Characteristics

Characteristic	Any forgone medical care (N = 1055)^a^			Missed dose of medicine (n = 725)			Missed scheduled medical care (n = 873)^b^		
	No./total No. (%)	95% CI, %	*P* value	No./total No. (%)	95% CI, %	*P* value	No./total No. (%)	95% CI, %	*P* value
Sex									
Men	234/458 (51)	44.9-57.1	[Reference]	41/299 (14)	9.0-20.4	[Reference]	211/384 (55)	48.5-61.5	[Reference]
Women	310/597 (52)	46.3-57.7	.81	66/426 (16)	10.3-22.9	.66	290/490 (59)	52.8-65.2	.38
Race/ethnicity									
White, non-Hispanic	356/697 (51)	46.1-56.0	[Reference]	50/482 (10)	7.0-15.2	[Reference]	337/600 (56)	50.8-61.4	[Reference]
Black, non-Hispanic	58/121 (48)	36.1-60.1	.65	18/81 (22)	11.3-37.7	.06	48/85 (56)	43.7-68.3	.98
Other, non-Hispanic	48/94 (51)	35.9-66.3	.99	7/52 (13)	2.8-43.0	.78	39/70 (57)	38.9-72.9	.96
Hispanic	82/143 (57)	45.1-69.0	.34	33/109 (30)	16.7-48.6	.004	77/119 (64)	50.7-76.0	.27
Age group									
≥65	125/262 (48)	41.4-54.2	[Reference]	10/160 (6)	3.4-11.4	[Reference]	119/231 (51)	44.5-58.1	[Reference]
50-64	155/276 (56)	48.9-62.6	.09	23/179 (13)	7.7-20.5	.08	146/242 (61)	52.9-67.6	.07
35-49	125/241 (52)	44.6-59.4	.39	29/182 (16)	10.3-24.5	.01	116/199 (58)	50.1-66.2	.19
18-34	139/276 (50)	39.3-61.6	.68	45/204 (22)	12.2-36.6	.004	120/202 (59)	45.8-71.7	.29
Household income, per y									
<$35 000	167/327 (51)	42.6-59.6	.94	66/244 (27)	18.0-38.3	.01	149/264 (56)	46.4-65.7	.96
$35 000-$74 999	177/344 (52)	44.1-58.9	[Reference]	26/226 (12)	6.6-19.6	[Reference]	163/288 (57)	49.1-63.8	[Reference]
≥$75 000	200/384 (52)	46.1-57.7	.93	16/255 (6)	3.7-9.9	.09	189/321 (59)	52.3-65.1	.65
Employment status									
Currently employed	251/503 (50)	43.9-55.9	[Reference]	46/367 (13)	8.4-18.7	[Reference]	225/405 (56)	49.0-61.9	[Reference]
Unemployed or not working owing to disability	121/186 (65)	55.2-73.7	.01	46/117 (39)	25.1-55.2	<.001	111/159 (70)	60.0-78.3	.02
Retired or providing unpaid family caregiving	129/271 (48)	41.3-54.2	.63	8/161 (5)	2.3-11.4	.05	125/239 (52)	45.3-59.1	.50
Insurance coverage									
Commercial or Medicare	387/768 (50)	46.1-54.8	[Reference]	52/517 (10)	7.0-14.0	[Reference]	360/645 (56)	51.1-60.3	[Reference]
Medicaid	86/142 (61)	44.4-75.4	.23	41/114 (36)	20.0-55.7	<.001	75/116 (65)	45.1-80.4	.37
Uninsured	45/88 (50)	35.4-65.5	.99	12/56 (21)	9.5-40.9	.09	40/61 (65)	48.3-79.3	.28

^a^ Forgone medical care includes missing 1 or more doses of a medicine the respondent typically picked-up or had someone else pick up from a retail pharmacy; missing a scheduled health care visit, including a preventive care visit, general medical outpatient visit, mental health outpatient visit, or elective surgical procedure; or not receiving care for a new severe (defined based on self report as severity 4-5 on a scale of 1-5) physical or mental health issue. Individuals could report multiple types of forgone care.

^b^ Scheduled medical care includes scheduled preventive care visits, scheduled general medical outpatient visits, scheduled mental health outpatient visits, and elective surgical procedures.

Respondents who were unemployed or not working owing to disability, compared with individuals who were employed, reported higher frequency of any forgone medical care (121 0f 186 respondents [65%] vs 251 of 503 respondents [50%]; *P* = .01), missed doses of prescription medication (46 of 117 respondents [39%] vs 46 of 367 respondents [13%]; *P* < .001), and missed scheduled medical care (111 of 159 respondents [70%] vs 225 of 405 respondents [56%]; *P* = .02). Compared with individuals with commercial health insurance or Medicare, those insured through Medicaid reported higher frequency of missed prescription medications (41 of 114 respondents [36%] vs 52 of 517 respondents [10%]; *P* < .001).

Frequency of forgone medical care varied by self-reported health status, number of prescription medications taken, and presence of a mental health condition (Table 2). Respondents who rated their health as fair or poor more often reported missing prescription medication compared with individuals who rated their health as excellent (35 of 149 respondents [24%] vs 5 of 41 respondents [11%]; *P* = .03), and those with 1 or more prescriptions reported forgoing any medical care less often than those with no prescription medication use (443 of 902 respondents [49%] vs 99 of 149 respondents [66%]; *P* = .005). Similarly, individuals with a mental health condition more often reported missing medication than individuals without a mental health condition (49 of 184 respondents [26%] vs 59 of 541 respondents [11%]; *P* = .004). No differences were detected in reported frequency of forgone medical care by other chronic health conditions examined, including heart disease, lung disease, or high blood pressure, diabetes, or high cholesterol.

**Table 2.** Share of Respondents Who Reported Needing Care Who Reported Forgone Medical Care From March Through Mid-July 2020, by Clinical Characteristics

Characteristic	Any forgone medical care (N = 1055)^a^			Missed dose of medicine (n = 725)			Missed scheduled medical care (n = 873)^b^		
	No./total No. (%)	95% CI, %	*P* value	No./total No. (%)	95% CI, %	*P* value	No./total No. (%)	95% CI, %	*P* value
Self-reported health									
Excellent	56/92 (61)	46.0-74.7	[Reference]	5/41 (11)	2.8-3.4	[Reference]	56/82 (69)	53.4-81.0	[Reference]
Very good	173/363 (48)	41.8-53.8	.11	25/254 (10)	5.7-16.3	.88	167/298 (56)	49.3-62.5	.13
Good	198/391 (51)	43.7-57.7	.21	43/281 (15)	9.6-23.1	.21	174/322 (54)	45.9-61.7	.09
Fair or poor	116/210 (56)	45.1-65.5	.54	35/149 (24)	13.0-39.4	.03	104/172 (61)	50.7-69.9	.36
Uses ≥1 prescription medications									
No	99/149 (66)	55.4-75.7	[Reference]	NA	NA	NA	95/145 (65)	54.1-75.0	[Reference]
Yes	443/902 (49)	44.7-53.6	.005	108/725 (15)	11.0-19.7	NA	404/725 (56)	50.8-60.6	.12
Has high blood pressure, diabetes, or high cholesterol									
No	337/650 (52)	46.1-57.6	[Reference]	71/433 (16)	10.8-23.8	[Reference]	309/519 (60)	53.1-65.7	[Reference]
Yes	207/405 (51)	45.4-56.7	.85	37/292 (13)	8.5-18.4	.37	192/354 (54)	48.0-60.1	.23
Has heart disease, such as a heart attack, coronary heart disease, angina, congestive heart failure, or other heart problems									
No	500/979 (51)	46.7-55.5	[Reference]	94/675 (14)	10.0-19.0	[Reference]	463/803 (58)	52.8-62.2	[Reference]
Yes	44/76 (58)	44.9-70.1	.33	13/50 (27)	13.0-47.4	.10	38/70 (55)	41.2-67.5	.69
Has lung disease, such as chronic bronchitis or emphysema									
No	515/1003 (51)	47.0-55.7	[Reference]	103/695 (15)	10.9-20.0	[Reference]	475/829 (57)	52.5-61.9	[Reference]
Yes	29/52 (56)	39.7-71.0	.59	4/30 (14)	5.8-29.4	.86	26/44 (59)	43.0-73.1	.85
≥1 Mental health conditions									
No	402/809 (50)	45.1-54.3	[Reference]	59/541 (11)	7.6-15.4	[Reference]	373/669 (56)	50.6-60.8	[Reference]
Yes	142/246 (58)	48.2-66.7	.13	49/184 (26)	16.3-39.9	.004	128/204 (63)	53.0-71.1	.22

^a^ Forgone medical care includes missing 1 or more doses of a medicine the respondent typically picked-up or had someone else pick up from a retail pharmacy; missing a scheduled health care visit, including a preventive care visit, general medical outpatient visit, mental health outpatient visit, or elective surgical procedure; or not receiving care for a new severe (defined based on self report as severity 4-5 on a scale of 1-5) physical or mental health issue. Individuals could report multiple types of forgone care.

^b^ Scheduled medical care includes scheduled preventive care visits, scheduled general medical outpatient visits, scheduled mental health outpatient visits, and elective surgical procedures.

**Figure 3.**
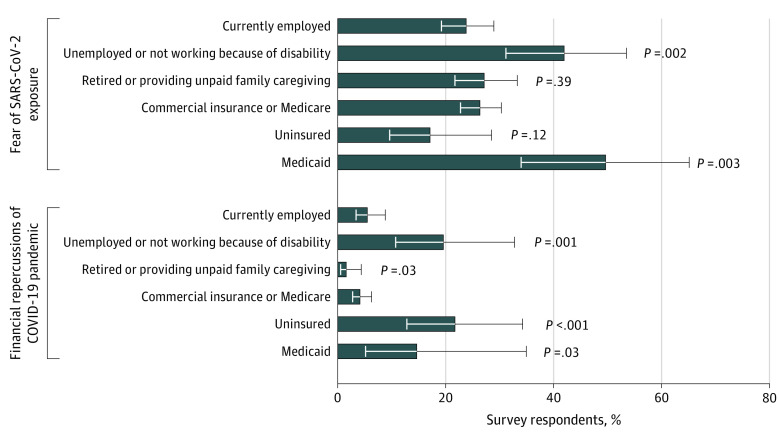
Reasons Reported for Forgoing Planned Care Among Respondents Who Reported Needing Care by Employment and Health Insurance Status Responses are based on the time period of March through mid-July 2020, during the initial phase of the coronavirus disease 2019 (COVID-19) pandemic in the United States. SARS-CoV-2 indicates severe acute respiratory syndrome coronavirus 2.

We identified differences in the reasons stated for forgoing medical care by employment and health insurance status (Figure 3). Compared with adults who were employed, adults who were unemployed more often attributed forgone medical care to fear of SARS-CoV-2 exposure (78 of 186 respondents [42%] vs 120 of 503 respondents [24%]; *P* = .002) and to financial repercussions of the pandemic (36 of 186 respondents [20%] vs 28 of 503 respondents [6%]; *P* = .001). Respondents without insurance reported forgoing medical care owing to financial concerns more often than respondents with commercial or Medicare health care coverage (19 of 88 respondents [22%] vs 32 of 768 respondents [4%]; *P* < .001). Respondents with Medicaid coverage, compared with respondents with commercial or Medicare coverage, more often reported forgoing care owing to concerns about SARS-CoV-2 exposure (70 of 142 respondents [50%] vs 203 of 768 respondents [26%]; *P* = .003) and financial concerns (21 of 142 respondents [15%] vs 32 of 768 respondents [4%]; *P* = .03). We also examined whether there were differences in reporting forgoing care owing to practice closures, but did not find statistically significant differences based on employment status or insurance coverage.

## Discussion

This survey study found that in a population representative of the overall US adult population, 41% of adults reported forgone care from March through mid-July 2020. Previous studies have found that individuals sometimes chose to forgo care prior to the COVID-19 pandemic; for example, the Kaiser Family Foundation estimated that in 2018, 13% of White individuals, 17% of Black individuals, and 21% of Hispanic individuals forwent care owing to cost.^12^ However, our results suggest that the COVID-19 pandemic exacerbated the problem, with individuals reporting closed practitioner offices, fear of exposure to SARS-CoV-2, and the financial repercussions of the pandemic as common reasons for forgoing care during this period.

These national survey results are consistent with research using insurance claims and EHR data that documented declines in the use of health care services to treat severe health issues during the first several months of the COVID-19 pandemic within specific health systems.^5,6,7^ Our results extend existing research on forgone medical care by quantifying changes at the national level, considering a larger set of health care services, and examining the underlying reasons reported for forgoing care during the initial phase of the pandemic.

The most common reason respondents reported for missing scheduled care was owing to office closure. The Coronavirus Aid, Relief, and Economic Security (CARES) Act^13^ included $175 billion to provide financial relief to medical practices and hospitals during the COVID-19 pandemic, and such funding may have helped practices that initially closed to reopen after putting additional safety precautions in place to prevent the spread of COVID-19. Proactive outreach from health care practitioner offices to reschedule cancelled appointments through in-person care or telehealth may help limit the long-term consequences of this forgone medical care. Telehealth can also help individuals continue to receive health care when they are concerned about exposure to SARS-CoV-2.^14^ States and the federal government have supported telehealth by temporarily loosening licensing, electronic prescribing, and written consent laws.^15,16,17^ Additionally, many payers have temporarily increased the types of services that can be delivered via telehealth and reimbursement for telehealth services.^18^ Continuing to provide financial and regulatory support for telehealth is important to ensure that practitioners offer this service for the duration of the pandemic. However, older adults who are uncomfortable with technology and individuals with limited internet connectivity may struggle to access or may be hesitant to use telehealth.^19^ It is important for practitioners and insurers to support patient use of telehealth and to ensure that telehealth can be accessed using a variety of internet speeds and devices, for example by offering audio-only (telephone) services.^20,21^

Among respondents who reported missing planned care, 14% reported the financial repercussions of the COVID-19 pandemic as a reason for forgoing care, and among the subset who reported missing prescription medication, nearly 1 in 4 respondents reported financial reasons for missing medications. Several policies can offer better financial protection to patients experiencing financial distress owing to the pandemic. Within the 38 states plus Washington, District of Columbia, that have expanded Medicaid, enrollment in Medicaid can improve health care affordability for individuals who have lost health insurance or were uninsured when the pandemic began. The $600 boost to weekly unemployment benefits during the first 4.5 months of the pandemic may have also mitigated some of the potentially harmful financial outcomes of the COVID-19 pandemic on people with health care needs. More individuals who are unemployed may forgo medical care as their unemployment benefits expire. Our results suggest that Medicare had a protective association, with older adults reporting much lower frequency of missed medication compared with other age groups. Conditioning businesses’ relief payments on keeping furloughed employees enrolled in their health insurance is another strategy that may prevent forgone care owing to cost concerns. Employers receiving federal assistance, such as the employee retention tax credit, are currently allowed, but not required, to pay for health insurance for furloughed employees.^22^

### Limitations

This study has several limitations. First, our sample size may have inhibited our ability to detect statistically significant differences in the frequency and reasons of forgone medical care, particularly when analyzing certain subgroups. Second, there may have been heterogeneity in responses to the COVID-19 pandemic owing to differences in timing and extent of the pandemic and public health responses in different locales not captured in our survey. Third, our survey items on forgone medical care were generated for this study, preventing us from directly comparing our findings with frequency of forgone medical care before the COVID-19 pandemic. Fourth, the AmeriSpeak panel used probability-based recruitment aligning with best-practice survey research standards, but results may be susceptible to sampling biases. Fifth, we did not have information on the employment or health insurance status of a respondent’s entire household. If a family member lost employment or health insurance owing to the pandemic, it could financially affect decision-making within the entire household about whether to seek or forgo care. Sixth, our analysis did not capture all types of forgone medical care; for example, we did not consider missed doses of mail-order drugs.

## Conclusions

The findings of this survey study suggest that as the United States is experiencing another wave of surging SARS-CoV-2 infections, it will be important to track whether interventions to enhance health system safety provide the public with sufficient confidence to seek medical care. As emergency financial measures enacted by the US Congress and unemployment benefits expire, ensuring the affordability of needed health care services for individuals financially impacted by COVID-19 is critical.

## Notes

Supplement. **eAppendix.** Question Wording for Forgone Medical Care, Health Insurance, and Employment Survey Questions **eTable.** Characteristics of Study Sample
