# Supplementary material for: Reports of Forgone Medical Care Among US Adults During the Initial Phase of the COVID-19 Pandemic
Source: JAMA Netw Open. 2021 Jan 21;4(1):e2034882. doi: 10.1001/jamanetworkopen.2020.34882 (PMC7821029; doi:10.1001/jamanetworkopen.2020.34882)
Supplement: Supplement. — eAppendix. Question Wording for Forgone Medical Care, Health Insurance, and Employment Survey Questions eTable. Characteristics of Study Sample [file jamanetwopen-e2034882-s001.pdf]

Supplemental Online Content

Anderson KE, McGinty EE, Presskreischer R, Barry CL. Reports of forgone medical care among US adults during the initial phase of the COVID-19 pandemic. *JAMA Netw Open*. 2021;4(1):e2034882. doi:10.1001/jamanetworkopen.2020.34882

eAppendix. Question Wording for Forgone Medical Care, Health Insurance, and Employment Survey Questions

eTable. Characteristics of Study Sample

This supplemental material has been provided by the authors to give readers additional information about their work.

© 2021 Anderson KE et al. *JAMA Network Open.*

eAppendix. Question Wording for Forgone Medical Care, Health Insurance, and Employment Survey Questions

1. In general, how would you rate your overall health?
    a. Excellent
    b. Very Good
    c. Good
    d. Fair
    e. Poor

2. Do you receive ongoing medical care for any of the following conditions? [select all that apply]
    a. High blood pressure (also known as hypertension), diabetes (also known as high blood sugar), or high cholesterol
    b. Heart disease, such as a heart attack, coronary heart disease, angina, congestive heart failure, or other heart problems
    c. Lung disease such as chronic bronchitis or emphysema
    d. Mental health conditions, such as depression or anxiety

3. Which of the following best describes how you received your prescription medications before the COVID-19 pandemic began in the U.S. in March 2020?
    a. I had typically picked-up my medication(s) from a pharmacy
    b. Someone else (e.g., relative, friend, neighbor) had typically picked up my medications for me
    c. I typically had my prescriptions delivered by home delivery or by mail
    d. I did not regularly take a prescription medication

Respondents were prompted to answer question 4 only if they selected option a for question 3.

4. Thinking about the medications that you had typically picked-up from the pharmacy, have you missed any doses during the COVID-19 pandemic?
    a. Yes
    b. No

Respondents were prompted to answer question 5 only if they selected option a for question 4.

5. What was the most important reason you missed doses?
    a. I did not want to risk exposure to COVID-19 by going to the pharmacy
    b. I was worried about the cost of paying for the medication due to the economic effects of COVID-19
    c. Another reason unrelated to COVID-19

Respondents were prompted to answer question 6 only if they selected option b for question 3.

6. Thinking about the medications that someone else typically picked-up from the pharmacy for you, have you missed any doses during the COVID-19 pandemic?
    a. Yes
    b. No

© 2021 Anderson KE et al. *JAMA Network Open.*

Respondents were prompted to answer question 7 only if they selected option a for question 6.

7. What was the most important reason you missed doses?
    a. I did not want the person that typically picks up my medications to risk exposure to COVID-19 by going to the pharmacy
    b. The person that typically picks up my medications did not want to risk exposure to COVID-19 by going to the pharmacy
    c. I was concerned about the cost of paying for the medication due to the economic effects of COVID-19
    d. Another reason unrelated to COVID-19

8. Since the COVID-19 pandemic began in the U.S. in March 2020, which of the following best describes any in-person <u>mental health-related appointments</u> you had scheduled? [select all that apply]
    a. I missed at least one appointment because I did not want to risk exposure to COVID-19
    b. I missed at least one appointment because I was worried about the cost of paying for the appointment due to the economic effects of COVID-19
    c. I missed at least one appointment because my provider's office was closed due to COVID-19
    d. I continued to go to my mental health-related appointments in person
    e. I continued to go to my mental health care-related appointments, but through telehealth (by phone or video)
    f. I did not have any mental health-related appointments scheduled during this period

9. Since the COVID-19 pandemic began in the U.S. in March 2020, which of the following best describes any in-person inpatient or outpatient <u>surgical procedures</u> you had scheduled? [select all that apply]
    a. I missed at least one surgical procedure because I did not want to risk exposure to COVID-19
    b. I missed at least one surgical procedure because I was worried about the cost of paying for the appointment due to the economic effects COVID-19
    c. I missed at least one surgical procedure because my provider's office was closed due to COVID-19
    d. I underwent my surgical procedure(s)
    e. I did not have any inpatient or outpatient surgical procedures scheduled during this period

10. Since the COVID-19 pandemic began in the U.S. in March 2020, which of the following best describes any in-person <u>preventive care appointments</u> you had scheduled? Preventive care includes screenings, immunizations, check-ups, and patient counseling to prevent illnesses, disease, or other health problems. [select all that apply]
    a. I missed at least one preventive care appointment because I did not want to risk exposure to COVID-19
    b. I missed at least one preventive care appointment because I was worried about the cost of paying for the appointment due to the economic effects of COVID-19
    c. I missed at least one preventive care appointment because my provider's office was closed due to COVID-19
    d. I continued to go to my preventive care appointments in person
    e. I continued to go to at least one preventive care appointment, but through telehealth (by phone or video)
    f. I did not have any preventive care visits scheduled during this period

© 2021 Anderson KE et al. *JAMA Network Open.*

11. Since the COVID-19 pandemic began in the U.S. in March 2020, which of the following best describes any in-person medical appointments (excluding preventive care, surgical procedures, and mental health appointments) you had scheduled? [select all that apply]
    a. I missed at least one appointment because I did not want to risk exposure to COVID-19
    b. I missed at least one appointment because I was worried about the cost of paying for the appointment due to the economic effects of COVID-19
    c. I missed at least one appointment because my provider's office was closed due to COVID-19
    d. I continued to go to my medical appointments in person
    e. I continued to go to medical appointments, but through telehealth (by phone or video)
    f. I did not have any medical appointments scheduled during this period

12. Did you have a new physical or mental health issue arise while social distancing guidelines were in place? [select all that apply]
    a. Yes, a physical health issue
    b. Yes, a mental health issue
    c. No

Respondents were prompted to answer questions 13 and 14 only if they selected option a for question 12.

13. How would you describe the severity of this physical health issue on a scale of 1 to 5, where 1 is a minor or non-urgent medical issue such as a headache and 5 is a severe, possibly life-threatening issue such as acute chest pain? If you had multiple medical issues arise, please answer this question and the following question based on the most severe issue that arose.
    a. 1
    b. 2
    c. 3
    d. 4
    e. 5

14. Which of the following best describes how you responded to this new physical health issue?
    a. I stayed at home and did not seek medical care
    b. I received medical care in person at a doctor's office, clinic, hospital, or other medical setting
    c. I received medical care through telehealth (by phone or video)
    d. I attempted to get treatment, but was unable to do so

Respondents were prompted to answer questions 15 and 16 only if they selected option b for question 12.

15. How would you describe the severity of this mental health issue on a scale of 1 to 5, where 1 is a minor or non-urgent issue such as passing feelings of anxiety and 5 is a severe issue such as major depression or anxiety that prevents you from carrying out daily activities for multiple days? If you had multiple mental health issues arise, please answer this question and the following question based on the most severe issue that arose.
    a. 1
    b. 2
    c. 3

© 2021 Anderson KE et al. *JAMA Network Open.*

    d. 4
    e. 5

16. Which of the following best describes how you responded to this new mental health issue?
    a. I stayed at home and did not seek medical care
    b. I received medical care in person at a doctor's office, clinic, hospital, or other medical setting
    c. I received medical care through telehealth (by phone or video)
    d. I attempted to get treatment, but was unable to do so

17. Are you now covered by any form of health insurance or health plan? A health plan would include any private insurance plan through your employer or a plan that you purchased yourself, as well as a government program like Medicare or Medicaid.
    a. Yes
    b. No

Respondents were prompted to answer question 18 only if they selected option a for question 17.

18. Which of the following is your main source of health insurance coverage?
    a. A plan through your employer
    b. A plan through your spouse's employer
    c. A plan you purchased yourself directly from an insurance company
    d. Marketplace
    e. Medicare
    f. Medicaid program
    g. Some other source

19. Which statement best describes your current employment status?
    a. Working full time for pay
    b. Working part time for pay
    c. Not working for pay – full or part time caregiver
    d. Not working for pay – on temporary layoff from a job
    e. Not working for pay – looking for work
    f. Not working for pay – retired
    g. Not working for pay – disabled
    h. Not working for pay – other

© 2021 Anderson KE et al. *JAMA Network Open.*

eTable. Characteristics of Study Sample

		Number of Respondents (%)
Sex		
	Male	646 (48%)
	Female	691 (52%)
Race/Ethnicity		
	White, non-Hispanic	840 (63%)
	Black, non-Hispanic	160 (12%)
	Other, non-Hispanic	115 (9%)
	Hispanic	223 (17%)
Age Group		
	18-34	394 (29%)
	35-49	323 (24%)
	50-64	333 (25%)
	65+	287 (21%)
Household Income		
	< $35,000	424 (32%)
	$35,000 - $74,999	429 (32%)
	≥ $75,000	485 (36%)
Employment Status		
	Currently Employed	678 (51%)
	Unemployed or Not Working Due to Disability	230 (17%)
	Retired or Providing Unpaid Family Caregiving	302 (23%)
	Unknown	128 (10%)
Insurance Coverage		
	Commercial or Medicare	930 (70%)
	Medicaid	166 (12%)
	Other Source of Insurance	63 (5%)
	Uninsured	168 (13%)
	Unknown	10 (1%)
Self-Reported Health		
	Excellent	146 (11%)
	Very Good	474 (35%)
	Good	478 (36%)
	Fair or Poor	239 (18%)
Takes One or More Prescription Medications		
	No	382 (29%)
	Yes	936 (70%)
	Unknown	20 (1%)
Has High Blood Pressure, Diabetes, or High Cholesterol		
	No	912 (68%)
	Yes	425 (32%)
Has Heart Disease, Such as a Heart Attack, Coronary Heart Disease, Angina, Congestive Heart Failure, or other Heart Problems		
	No	1,258 (94%)
	Yes	79 (6%)
Has Lung Disease, Such as Chronic Bronchitis or Emphysema		
	No	1,281 (96%)
	Yes	56 (4%)
Has One or More Chronic Health Conditions		

© 2021 Anderson KE et al. *JAMA Network Open.*

		Number of Respondents (%)
	No	864 (65%)
	Yes	473 (35%)
Has One or More Mental Health Conditions		
	No	1,058 (79%)
	Yes	279 (21%)

© 2021 Anderson KE et al. *JAMA Network Open.*
